# Genomic Sequencing of SARS-CoV-2 E484K Variant B.1.243.1, Arizona, USA

**DOI:** 10.3201/eid2710.211189

**Published:** 2021-10

**Authors:** Peter T. Skidmore, Emily A. Kaelin, LaRinda A. Holland, Rabia Maqsood, Lily I. Wu, Nicholas J. Mellor, Joy M. Blain, Valerie Harris, Joshua LaBaer, Vel Murugan, Efrem S. Lim

**Affiliations:** Arizona State University, Tempe, Arizona, USA

**Keywords:** COVID-19, coronavirus disease, SARS-CoV-2, severe acute respiratory syndrome coronavirus 2, viruses, respiratory infections, zoonoses, emergent variant, genomic surveillance, Arizona, United States

## Abstract

Genomic surveillance can provide early insights into new circulating severe acute respiratory syndrome coronavirus 2 (SARS-CoV-2) variants. While conducting genomic surveillance (1,663 cases) from December 2020–April 2021 in Arizona, USA, we detected an emergent E484K-harboring variant, B.1.243.1. This finding demonstrates the importance of real-time SARS-CoV-2 surveillance to better inform public health responses.

Genomic sequencing surveillance tracks the evolution of severe acute respiratory syndrome coronavirus 2 (SARS-CoV-2) and can provide early-warning insight of new variants circulating in communities. SARS-CoV-2 continues to acquire mutations in its genome as it spreads around the world. Although many mutations have little or no consequence on virus fitness, some mutations affect receptor binding or reduce antibody neutralization (*1*,*2*). Other mutations have been associated with increased transmission and clinical disease severity (*3*; Y. Liu et al., unpub. data, https://doi.org/10.1101/2021.03.08.434499). As of July 2021, the US SARS-CoV-2 Interagency Group has designated 4 variants of concern (VOC) and 7 variants of interest (VOI) in the United States based on the combination of mutations and associated attributes (*5*). Several of these VOCs and VOIs (e.g., Beta/B.1.351, Gamma/P.1, Delta/B.1.617.2) harbor the E484K mutation in the spike glycoprotein gene (*4*). Studies have demonstrated that the E484K mutation reduces antibody neutralization (*2*,*5*,*6*). E484K variants have also been identified in reinfection cases, suggesting a role in breakthrough infections (*2*,*5*–*7*); these findings indicate the need to monitor for SARS-CoV-2 variants in real time.

In an effort to provide statewide genomic surveillance, we sequenced the SARS-CoV-2 genome from 1,663 positive samples collected December 28, 2020–April 12, 2021 in Arizona, United States. Samples were primarily from Maricopa (56.9%), Coconino (26.4%), and Pima (8.5%) Counties. Study participants were 53.8% male, 46.2% female; age range was 5–81 years (median of 25 years). We successfully sequenced 1,538 (92.5%) high-quality complete genomes and found VOCs Alpha/B.1.1.7 (n = 336, 21.8%), Gamma/P.1 (n = 5, 0.33%), Beta/B.1.351 (n = 1, 0.07%), and Delta/B.1.617.2 (n = 1, 0.07%) and VOIs Epsilon/B.1.427/B.1.429 (n = 416, 27.0%), Iota/B.1.526 (n = 7, 0.5%), and Zeta/P.2 (n = 8, 0.5%) (Appendix Table 1). We detected 8 genomes associated with a common B.1.243 variant that had acquired an E484K mutation in the spike protein. The novel variant had 11 lineage-defining mutations, including V213G and E484K in the spike gene, a 9-nt deletion in open reading frame (ORF) 1ab (ΔSGF3675–77), a 3-nt insertion in the noncoding intergenic region upstream of the N gene, and other synonymous substitutions (Appendix Table 2, Figure 1). These 11 conserved mutations are distinct from the mutations associated with the parent lineage, B.1.243. The parent B.1.243 lineage is a common variant circulating in the United States that was observed in March 2020, early in the pandemic (Figure, panels A–C). The B.1.243 parent lineage encodes the spike gene D614G substitution but none of the other concerning mutations (Appendix Table 3, Figure 1). This new E484K-harboring variant has been officially designated as B.1.243.1 using the pangolin nomenclature system (*8*).

**Figure:**
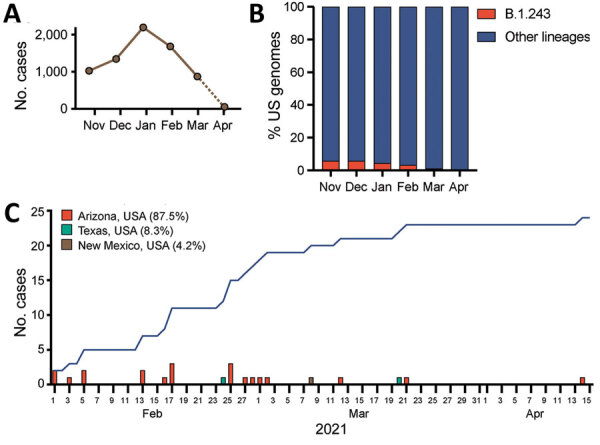
Emergence of E484K-harboring B.1.243.1 variant of severe acute respiratory syndrome coronavirus 2 (SARS-CoV-2) in Arizona, United States. A) Prevalence of B.1.243 parental lineage in the United States by number of cases per month, November 2020–April 2021. Dashed line indicates incomplete reporting of sequences from April 2021. B) Prevalence of B.1.243 parental lineage in the United States by proportion of sequenced genomes per month, November 2020–April 2021. C) Total B.1.243.1 cases reported February–April 2021. Blue curve indicates cumulative case incidence.

We examined the GISAID repository (https://www.gisaid.org) for additional B.1.243.1 genomes to determine its prevalence and geographic distribution. We found that B.1.243.1 is predominantly established in Arizona. Of 24 cases of B.1.243.1 sequenced during February 1–April 14, a total of 21 cases were from Arizona (Figure, panel C; Appendix Table 4). Two cases were sequenced from samples collected in Texas on February 24 and March 20 and another from a sample collected in New Mexico on March 8, suggesting that B.1.243.1 had spread to other states. We also identified 2 instances in which the parent B.1.243 lineage independently acquired the E484K mutation. However, both genomes lacked the other B.1.243.1 lineage-defining mutations and appear to be dead-end transmission events. Phylogenetic analyses indicate that the B.1.243.1 sequences form a monophyletic clade within the B.1.243 clade (Appendix Figure 2). Multiple internal branching observed in the B.1.243.1 clade indicates continued diversification of the lineage sequences, which suggests that B.1.243.1 was being established in circulation within Arizona. In contrast, the 2 additional B.1.243 cases bearing the E484K mutation alone were phylogenetically distinct from the B.1.243.1 clade, suggesting that those isolates had evolved independently.

Genomic sequencing surveillance can provide early warnings of emergent variants. Because phylogenetic evidence suggested that B.1.243.1 was beginning to circulate in Arizona, the Arizona Department of Health Services (ADHS) was notified on March 18, 2021, and contact tracing was performed for the early B.1.243.1 cases. Of the case-patients who were interviewed, none reported connection to other patients. At the time of reporting (May 2021), the most recent case of B.1.243.1 had been reported on April 14, 2021 (Appendix Table 4). The limited spread of B.1.243.1 coincides with competition from the rapid rise in transmission of the Alpha (B.1.1.7) variant in the United States (*9*). 

A limitation of this study is that the sequencing surveillance represented 0.31% of 503,825 total SARS-CoV-2 cases in Arizona during the study period. Targeted sampling efforts, such as prescreening samples for the E484K mutation by PCR-based assays, would complement random sampling for genomic sequencing surveillance. Our study highlights the need for sustained genomic surveillance in public health strategies and responses.

Appendix Additional information about SARS-CoV-2 E484K variant B.1.243.1, Arizona, United States.
