# Supplementary material for: Genomic Sequencing of SARS-CoV-2 E484K Variant B.1.243.1, Arizona, USA
Source: Emerg Infect Dis. 2021 Oct;27(10):2718–20. doi: 10.3201/eid2710.211189 (PMC8462350; doi:10.3201/eid2710.211189)
Supplement: Appendix — Additional information about SARS-CoV-2 E484K variant B.1.243.1, Arizona, United States. [file 21-1189-Techpp-s1.pdf]

Article DOI: https://doi.org/10.3201/eid2710.211189

Genomic Sequencing of SARS-CoV-2 E484K Variant B.1.243.1, Arizona, USA

Appendix

Materials and Methods

Study Population

As part of severe acute respiratory syndrome coronavirus 2 (SARS-CoV-2) genomic surveillance efforts, saliva samples submitted for COVID-19 testing to the Arizona State University's Biodesign Clinical Testing Laboratory (ABCTL) that tested positive (TaqPath COVID-19 Combo Kit, Applied Biosystems, https://www.thermofisher.com) were randomly selected for next-generation sequencing (*1*). Samples covered a broad distribution from counties across Arizona, United States. The Arizona State University Institutional Review Board approved this study.

SARS-CoV-2 Sequencing

RNA was extracted from 250 µL of saliva sample (KingFisher Flex, Thermo Scientific) according to the manufacturer's guidelines. First-strand cDNA synthesis was performed using random hexamers (SuperScript III/IV reverse transcription; Life Technologies, https://www.thermofisher.com), followed by PCR amplification of tiled amplicons spanning the SARS-CoV-2 genome (Swift Normalase Amplicon Panel; Swift Biosciences, https://swiftbiosci.com) and library construction. Libraries were sequenced on the Illumina MiSeq (version 2, 2×150; https://www.illumina.com) and NextSeq 500 (version 2.5, 2×150, mid or high output).

Sequencing Analysis

Illumina sequencing reads were quality filtered to remove adaptors and low-quality bases using BBTools (https://jgi.doe.gov/data-and-tools/bbtools). High-quality-filtered reads were mapped to the SARS-CoV-2 Wuhan1 reference genome (NC_045512.2) using BWA-MEM (H. Li, unpub. data, https://arxiv.org/abs/1303.3997) and amplicon primers were trimmed using

Primerclip version 0.3.8 (*2*). Consensus sequences were called using iVar (version 1.0; parameters -q 20, -t 0.75, -m 20, -n N) (*3*). Lineages were assigned using pangolin version 2.3.8 (*4*). Sequence alignments were performed with MAFFT version 7.471 (*5*) and variant calling using Geneious Prime version 2021 (https://www.geneious.com). High-quality complete genomes were defined as genomes >29,000 bp in length with <50% ambiguities. Sequences used in phylogenetic analysis include the global nextregions sequences from GISAID (*6*) subset to 500 randomly selected sequences and a random subset of 100 B.1.243 sequences from all B.1.243 GISAID sequences using the augur filter command (–no-probabilistic-sampling) from NextStrain (https://docs.nextstrain.org), and the 24 B.1.243.1 lineage sequences. Phylogenetic reconstruction was performed with IQTree version 2.0.3 (*7*), iqtree -nt AUTO -bb 1000 -m MFP -mset GTR, and Augur version 11.3.0 (*8*).

Data Availability

Sequence data have been deposited in GISAID.

References

1. Holland LA, Kaelin EA, Maqsood R, Estifanos B, Wu LI, Varsani A, et al. An 81-nucleotide deletion in SARS-CoV-2 ORF7a identified from sentinel surveillance in Arizona (January to March 2020). J Virol. 2020;94:e00711-20. PubMed https://doi.org/10.1128/JVI.00711-20

2. Swift. primerclip. 2021 [cited 2021 Jul 22]. https://github.com/swiftbiosciences/primerclip

3. Grubaugh ND, Gangavarapu K, Quick J, Matteson NL, De Jesus JG, Main BJ, et al. An amplicon-based sequencing framework for accurately measuring intrahost virus diversity using PrimalSeq and iVar. Genome Biol. 2019;20:8. PubMed https://doi.org/10.1186/s13059-018-1618-7

4. O'Toole A, Scher E, Underwood A, Jakcson B, Hill V, McCrone JT, et al. pangolin. [cited 2021 Jul 22]. https://github.com/cov-lineages/pangolin

5. Katoh K, Misawa K, Kuma K, Miyata T. MAFFT: a novel method for rapid multiple sequence alignment based on fast Fourier transform. Nucleic Acids Res. 2002;30:3059–66. PubMed https://doi.org/10.1093/nar/gkf436

6. Elbe S, Buckland-Merrett G. Data, disease, and diplomacy: GISAID's innovative contribution to global health. Glob Chall. 2017;1:33–46. PubMed https://doi.org/10.1002/gch2.1018

7. Minh BQ, Schmidt HA, Chernomor O, Schrempf D, Woodhams MD, von Haeseler A, et al. IQ-TREE 2: new models and efficient methods for phylogenetic inference in the genomic era. Mol Biol Evol. 2020;37:1530–4. PubMed https://doi.org/10.1093/molbev/msaa015

8. Huddleston J, Hadfield J, Sibley TR, Lee J, Fay K, Ilcisin M, et al. Augur: a bioinformatics toolkit for phylogenetic analyses of human pathogens. J Open Source Softw. 2021;6:2906. PubMed https://doi.org/10.21105/joss.02906

Appendix Table 1. SARS-CoV-2 lineages found in general surveillance sequencing of 1,538 positive samples, Arizona*

Lineage	Count	Lineage	Count	Lineage	Count
A.2.4	1	B.1.177	1	B.1.429	289
A.2.5	1	B.1.189	3	B.1.517	1
B	2	B.1.2	327	B.1.526	7
B.1	86	B.1.232	3	B.1.526.1	2
B.1.1	11	B.1.234	13	B.1.526.2	4
B.1.1.1	3	B.1.239	8	B.1.551	12
B.1.1.142	1	B.1.240	2	B.1.561	12
B.1.1.207	1	B.1.241	1	B.1.564	1
B.1.1.222	12	B.1.243	50	B.1.567	1
B.1.1.231	1	B.1.243.1	1	B.1.568	3
B.1.1.239	1	B.1.265	1	B.1.575	1
B.1.1.28	1	B.1.311	11	B.1.577	1
B.1.1.316	6	B.1.336	2	B.1.582	1
B.1.1.318	2	B.1.346	1	B.1.595	3
B.1.1.322	1	B.1.351	1	B.1.596	49
B.1.1.348	3	B.1.369	2	B.1.609	11
B.1.1.416	1	B.1.375	2	B.1.612	1
B.1.1.432	3	B.1.378	1	B.1.617.2	1
B.1.1.519	64	B.1.396	2	B.1.81	1
B.1.1.7	336	B.1.400	6	C.13	1
B.1.111	1	B.1.404	12	P.1	5
B.1.153	2	B.1.423	1	P.2	8
B.1.160	1	B.1.427	127	R.1	4

*SARS-CoV-2, severe acute respiratory syndrome coronavirus 2.

Appendix Table 2. B.1.243.1 lineage–defining mutations in SARS-CoV-2 samples, Arizona*

Gene	Nucleotide change	Amino acid change	Number of B.1.243.1 genomes with mutation
ORF1ab	C4321T	Synonymous	24/24
ORF1ab	11288–11296 deletion	SGF 3675–3677 deletion	24/24
ORF1ab	C17999T	T5912I	24/24
ORF1ab	G19962T	Synonymous	24/24
S	T22200G	V213G	23/23†
S	G23012A	E484K	22/22†
M	C26873T	Synonymous	24/24
M	G27065A	Synonymous	24/24
Noncoding, upstream of N	28266 GCC insertion	Non-coding	24/24
N	C28603T	Synonymous	24/24
3′ UTR	29750–29761 deletion	Non-coding	19/19†

* All genome positions in reference to the SARS-CoV-2 Wuhan-1 sequence (NC_045512.2). ORF, open reading frame; SARS-CoV-2, severe acute respiratory syndrome coronavirus 2.
†1 or more sequences omitted due to ambiguous (N) nucleotides or low coverage.

Appendix Table 3. Lineage-defining mutations in SARS-CoV-2 samples of parental B.1.243 lineage, Arizona

Gene	Nucleotide change	Amino acid change	% of B.1.243 genomes with mutation
5′ UTR	C241T	Non-coding	98.0
ORF1ab	C3037T	Synonymous	99.4
ORF1ab	C14408T	P4715L	98.6
ORF1ab	A20268G	Synonymous	95.5
S	A23403G	D614G	100
S	T24076C	Synonymous	98.9
N	C28854T	S194L	97.9

*Based on 7,211 global B.1.243 genomes downloaded from GISAID on March 20, 2021. ORF, open reading frame; SARS-CoV-2, severe acute respiratory syndrome coronavirus 2.

Appendix Table 4. B.1.243.1 sequences reported in study of SARS-CoV-2 mutations, Arizona*

Name	GISAID accession no.	State	County	Collection date	ORF1ab C_t	S C_t	N C_t
hCoV-19/USA/AZ-ASU2621/2021	EPI_ISL_1364812	Arizona	Pima	2021 Feb 1	26.2	26.3	26.8
hCoV-19/USA/AZ-ASU2625/2021	EPI_ISL_1364644	Arizona	Pima	2021 Feb 1	27.2	27.4	27.8
hCoV-19/USA/AZ-ASU2857/2021	EPI_ISL_1364649	Arizona	Pima	2021 Feb 3	23.1	23.1	22.7
hCoV-19/USA/AZ-CDC-21801839/2021	EPI_ISL_1090700	Arizona	NA	2021 Feb 5	NA	NA	NA
hCoV-19/USA/AZ-CDC-21802041/2021	EPI_ISL_1090853	Arizona	NA	2021 Feb 5	NA	NA	NA
hCoV-19/USA/AZ-CDC-22062741/2021	EPI_ISL_1139766	Arizona	NA	2021 Feb 13	NA	NA	NA
hCoV-19/USA/AZ-ASU2754/2021	EPI_ISL_1364775	Arizona	Maricopa	2021 Feb 13	14.7	14.8	14.9
hCoV-19/USA/AZ-ASU3758/2021	EPI_ISL_1592344	Arizona	Maricopa	2021 Feb 16	28.9	30	28.1
hCoV-19/USA/AZ-ASU3132/2021	EPI_ISL_1365543	Arizona	Maricopa	2021 Feb 17	20.7	20	21.2
hCoV-19/USA/AZ-ASU2925/2021	EPI_ISL_1365483	Arizona	Maricopa	2021 Feb 17	28	26.8	28
hCoV-19/USA/AZ-ASU3099/2021	EPI_ISL_1365622	Arizona	Maricopa	2021 Feb 17	24.1	23.2	24
hCoV-19/USA/TX-HMH-MCoV-29140/2021	EPI_ISL_1303700	Texas	Harris	2021 Feb 24	NA	NA	NA
hCoV-19/USA/AZ-ASU2540/2021	EPI_ISL_1291671	Arizona	Maricopa	2021 Feb 25	26	26.4	27
hCoV-19/USA/AZ-TG758899/2021	EPI_ISL_1292269	Arizona	Maricopa	2021 Feb 25	NA	NA	NA
hCoV-19/USA/AZ-TG758666/2021	EPI_ISL_1292117	Arizona	Maricopa	2021 Feb 25	NA	NA	NA
hCoV-19/USA/AZ-CDC-22555310/2021	EPI_ISL_1290985	Arizona	NA	2021 Feb 27	NA	NA	NA
hCoV-19/USA/AZ-TG759060/2021	EPI_ISL_1292381	Arizona	Maricopa	2021 Feb 28	NA	NA	NA
hCoV-19/USA/AZ-CDC-22554229/2021	EPI_ISL_1290992	Arizona	NA	2021 Mar 1	NA	NA	NA
hCoV-19/USA/AZ-TG761699/2021	EPI_ISL_1296905	Arizona	NA	2021 Mar 2	NA	NA	NA
hCoV-19/USA/NMDOH-2021075279/2021	EPI_ISL_1340909	New Mexico	NA	2021 Mar 8	NA	NA	NA
hCoV-19/USA/AZ-TG787352/2021	EPI_ISL_1464762	Arizona	Maricopa	2021 Mar 12	NA	NA	NA
hCoV-19/USA/TX-CDC-QDX23213780	EPI_ISL_1479977	Texas	NA	2021 Mar 20	NA	NA	NA
hCoV-19/USA/AZ-CDC-QDX23313079/2021	EPI_ISL_1525953	Arizona	NA	2021 Mar 21	NA	NA	NA
hCoV-19/USA/AZ-CDC-ASC210070999/2021	EPI_ISL_1999732	Arizona	NA	2021 Apr 14	NA	NA	NA

*C_t, cycle threshold; NA, not available; ORF, open reading frame; SARS-CoV-2, severe acute respiratory syndrome coronavirus 2.

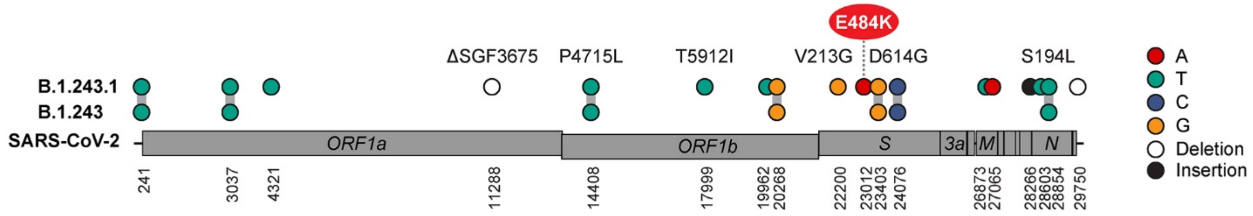

Appendix Figure 1. B.1.243.1 lineage-defining mutations on the severe acute respiratory syndrome coronavirus 2 (SARS-CoV-2) genome. Mutations are shown in reference to the SARS-CoV-2 Wuhan-1 genome position (NC_045512.2). ORF, open reading frame.

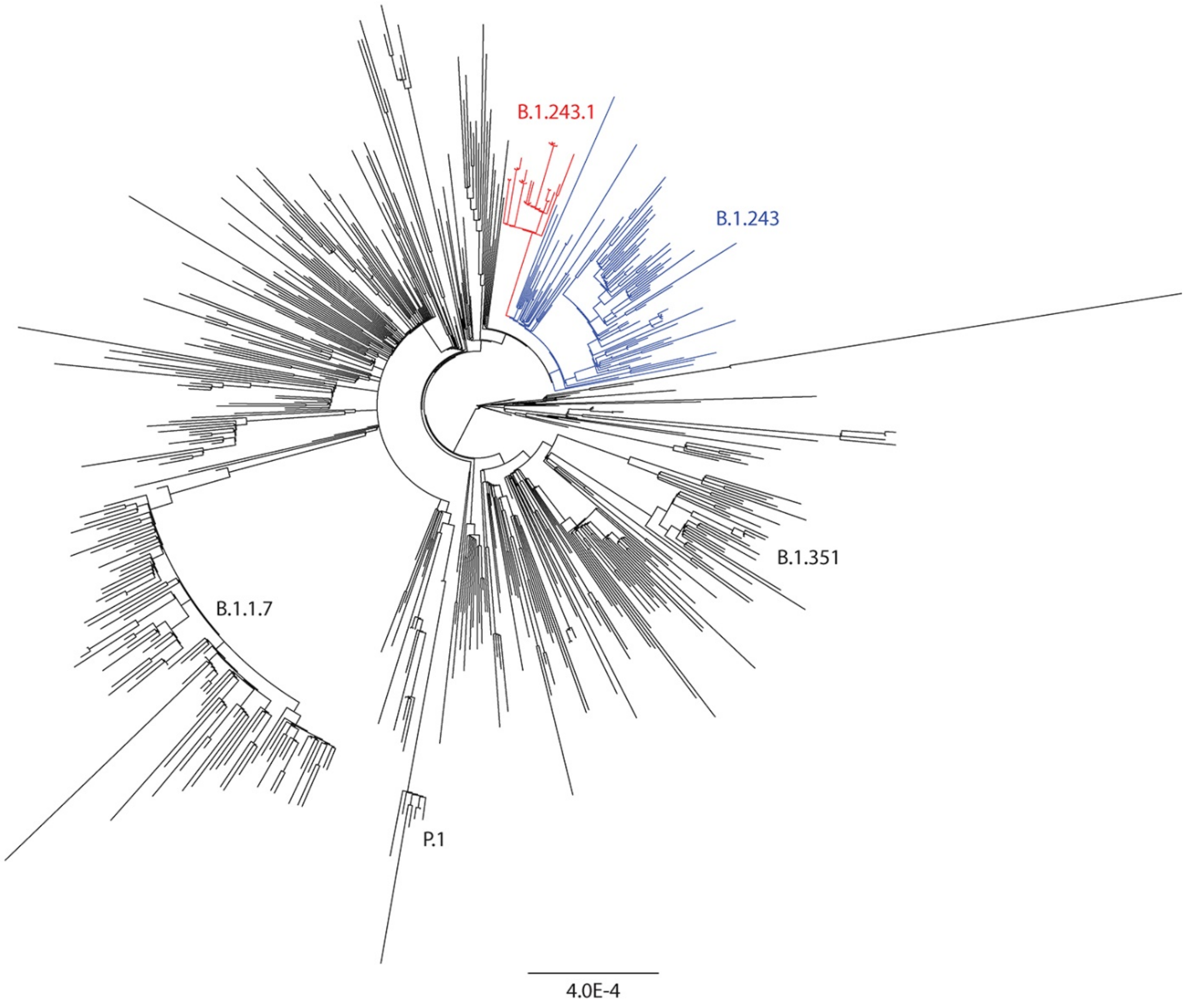

Appendix Figure 2. Maximum-likelihood phylogeny of diverse SARS-CoV-2 sequences including 500 representative global sequences, 100 B.1.243 parent lineage sequences, and the 24 B.1.243.1 sequences we identified. The novel B.1.243.1 lineage is indicated in red branches (clade bootstrap support: 100), and the parental B.1.241 lineage in blue. Scale bar represents number of nucleotide substitutions per site.
